# Editorial: Evaluation of preharvest and postharvest factors on forage crop quality, physiology, and ensiling characteristics

**DOI:** 10.3389/fpls.2024.1421788

**Published:** 2024-05-20

**Authors:** Siran Wang, Qing Zhang, Lin Sun, Huili Pang, Ping Li, Nazir Ahmad Khan

**Affiliations:** ^1^ Institute of Animal Science, Jiangsu Academy of Agricultural Science, Nanjing, China; ^2^ Key Laboratory of Crop and Animal Integrated Farming, Ministry of Agriculture, Jiangsu Academy of Agricultural Science, Nanjing, China; ^3^ College of Forestry and Landscape Architecture, South China Agricultural University, Guangzhou, China; ^4^ Inner Mongolia Engineering Research Center of Development and Utilization of Microbial Resources in Silage, Inner Mongolia Academy of Agriculture and Animal Husbandry Science, Hohhot, China; ^5^ Henan Provincial Key Laboratory of Ion Beam Bio-Engineering, School of Agricultural Sciences, Zhengzhou University, Zhengzhou, China; ^6^ College of Animal Science, Guizhou University, Guiyang, China; ^7^ Department of Animal Nutrition, The University of Agriculture Peshawar, KP, Peshawar, Pakistan

**Keywords:** forage, microbial community, nutritive value, ensiling, silage, crop management

The purpose of making silage is to preserve the nutritive value of the harvested crop. The preharvest factors of forages that affect silage nutritional value are plant physical structure and chemical composition, which are mainly affected by maturity at harvest, plant genotypes (species, cultivars within species, hybrids), crop management (sowing time, planting method, irrigation, fertilization, weeds and pest control, plant microbiology), epiphytic and endophytic microbiota (particularly lactic acid producing bacteria (LAB)), and the environment in which the crop is planted and harvested (temperature, rainfall, weather, soil, day light, and some nature disasters). The postharvest factors include harvest management (machinery, cutting height, wilting, bruising and chopping), ensiling management (type of silo, compaction/density, sealing, type and dose of silage additives) and feeding management (exposure time to air). Silo management is also a key factor that affects quality of ensiled forages, including the speed and degree of filling silo, the storage method, and the rate of consuming silage. Recently, most researchers focused on the improvement of forage yield and fermentation quality through breeding, optimizing crop harvest maturity, and using various biological and chemical additives for improving silage fermentation quality and post-ensiling stability. However, the aforementioned factors are often not integrated with field management and methods of harvesting and ensiling in improving silage fermentation and nutritive value. Hence, this Research Topic aimed to explore the preharvest and postharvest factors affecting forage physiology, nutritional quality, fermentation characteristics and post-ensiling quality.

Our Research Topic comprises 8 original research articles and 2 reviews contributed by 67 authors. In this topic, Wang et al. (2024) evaluated the effects of adding *Bacillus velezensis* to whole-plant corn silage (WPCS), to explore the factors contributing to the fermentation characteristics of WPCS. They found that *B. velezensis* enhanced the silage quality and aerobic stability of WPCS during the milk-ripe stage by promoting the proliferation of LAB. In comparison to *Lactiplantibacillus plantarum*, *B. velezensis* rapidly lowered the pH of WPCS, caused greater degradation of fiber compositions, and improved water-soluble carbohydrate (WSC) level, and aerobic stability of the silage.

Crop–livestock integrated systems can optimize the efficiency of inputs and nutrients (re)-utilization, and promote the production and sustainability in agriculture. Nevertheless, limited studies investigated the triple intercropping of forages for ensiling. Prado et al. (2023) evaluated the dry biomass yield, ensiling characteristics, and nutritional components of sorghum silage intercropped with *Stylosanthes* cv. Bela and *Tamani guinea* grass as an integrated cropping system for silage production. The results indicated intercropping of sorghum with tropical forages could be used for producing mixed silage with several advantages, such as greater biomass production per unit area, superior nutritional quality, and pasture availability after harvesting crop for grazing and producing silage, finally improving the land-use efficiency with a sustainable way. Mixed silage from sorghum with *Stylosanthes* cv. Bela and *Tamani guinea* grass presented better fermentation quality, higher total digestible nutrient and ether extract contents than monocropped-forage silage. Mixed cropping with tropical forages increased the crude protein (CP) content as compared to monocropped-sorghum silage, which is expected to probably decrease costs with acquisition of protein supplements. Finally, they suggested the triple intercropping of Bela, sorghum, and *Tamani guinea* grass for producing mixed silage, due to higher yield, better fermentation and nutritional value of silage, and fresh forage availability for a longer period.

The WPCS is an important feedstuff of dairy rations globally whereby its great biomass yield and metabolizable energy. Whereas, the nutritive value of WPCS changed in different seasons during the developing stage. The interactions among management (M) × environment (E) × genotype (G) influenced the partitioning to grain (Harvest Index, HI). Hence, modelling tool could be used to accurately predict the variations of HI during crop growth. Ojeda et al. (2023) conducted a study to (i) explore the main factors affecting HI variability and grain yield, (ii) check the APSIM system (Agricultural Production Systems Simulator) to evaluate the growth of crop and crop partitioning with sufficient experimental data, and (iii) study the main resources of HI difference with a large scale of combined M × E × G. Sowing date, nitrogen rates, plant density, harvest date, genotype data, and irrigation rates were utilized from experimental fields to evaluate the main factors of HI variability and to check the growth of corn with APSIM. They concluded that the model supplies some good insights into the improvement of nutritional value of corn silage, and choosing the genotype and harvest timing when making decision.

Some plants could tolerate the poor soil and drought and high-temperature conditions, thus they could be utilized as feed sources for ruminants under different harsh environments. Amaranth (*Amaranthus hypochondriacus*) is one of such plants. Furthermore, a lower content of lignin (4% DM) and lower levels of nitrate and oxalic acid, and a higher content of CP (25% DM) were found in amaranth compared to corn. Ma et al. (2023) selected LAB, glucose and cellulase as silage additives, and investigated the impacts of various additives on the ensiling characteristics, ruminal digestion, and aerobic stability of mixed silages containing corn straw and amaranth. They found that the combined addition of cellulose, glucose, and LAB enhanced CP, lactic acid, DM contents and LAB populations, and reduced neutral detergent fiber (NDF) and acid detergent fiber (ADF) contents. The combined inoculant also decreased mold and aerobic bacteria population and enhanced ruminal digestion and aerobic stability of mixed silages containing corn straw and amaranth.

The saline–alkali soils are widely distributed in the world. Alfalfa (*Medicago sativa* L.) is a good legume forage with high palatability and nutritive value. The rood system of alfalfa is very deep, contributing to fixing the nitrogen, thus planting alfalfa can improve the soil fertility. In saline–alkali soils, alfalfa could supply a large amount of quality protein, with ensiling being one of good utilization and preservation ways, especially in some fields with climatic limitations. Shi et al. (2023) evaluated the effects of endogenous potassium (K^+^) and sodium (Na^+^) in crops on the silage quality and bacterial community compositions during the ensiling of alfalfa. The results showed that the level of Na^+^ in silage enhanced as the salt stress increasing. Enhanced salt stress resulted in changes in bacterial compositions, with increased abundances of *Pantoea* and *Lactococcus*, particularly under high salt stress. Moreover, the accumulation of endogenous Na^+^ in alfalfa limited bacterial growth under salt stress, thus suppressing protein degradation during ensiling. Higher levels of Na^+^ can be found in alfalfa silage under higher salt stress. The good silage quality of alfalfa under salt stress could be ascribed to the dominant genera *Lactobacillus* and *Lactococcus* in alfalfa silage.

Corn often has lower counts of epiphytic LAB, and contents of lactic acid and WSC contents, and poor aerobic stability and silage quality in high-temperature environment. Khan et al. (2023) systematically assessed the impacts of molasses, homofermentative LAB (homLAB), heterofermentative LAB (hetLAB) and their mixture (MIX) on fermentative profile, chemical compositions, carbohydrate fractions, *in vitro* digestibility of DM (IVDMD), microbial populations, and aerobic stability of corn in high-temperature environment (30~45°C). They concluded that applying additives could enhance DM recovery, nutritive value, silage quality, and aerobic stability of corn under high-temperature condition. The MIX inoculant also performed well in improving the silage quality and aerobic stability, whereas more studies are required, especially in the effect of dose rate.

The level of STC (kernel starch) can indicate the nutritive value of corn, and is directly related to the grains’ aroma and taste. The magnesium (Mg) and calcium (Ca) are important nutrients for the development and growth of corn and the STC content. To assess the physiological mechanisms of Mg and Ca impacts on the production of STC in corn kernel and impacts of endogenous enzymes and hormones synthesis in corn leaves on STC, He et al. (2024) added foliar Mg and Ca fertilizers at different doses to corn before pollination. They reported that the production of STC was evidently increased by Mg and Ca addition with modulating the levels of endogenous hormone and activity of key enzymes. Their results explore a new way to enhance the nutritive value of corn.

Recently, volatile metabolomics and microbiomics have become important tools of modern biotechnology, showing potential for more applications in food nutrition and science field. Microbiomics gives some insights into the abundance, species, and functionality of various microbes with studying the microbial community, which can also present the correlations with the flavor and quality of fermentative products. Volatile metabolomics could be used to identify the relationship between microbial activity and fermentation products via studying the variation and compositions of volatile compounds in foods. Deng et al. (2023) analyzed the silage quality, volatile metabolites, and bacterial communities of oat silages harvested at two growth stages, and examined the relationship between volatile metabolites and microbes. They concluded that the growth stages had great impacts on nutritional compositions, fermentative products, and flavor characteristics of oat, with the fermentation profile predominated by *Lactiplantibacillus* resulting in favorable flavor, while the fermentation profile predominated by *Enterococcus* resulted in unpalatable flavor.

In poultry and livestock production, some plant extracts can be used as additives to replace the antibiotics. Plant extracts are extracted from plants, and contain some bioactive components and pharmacological property, thus they need to be further studied. Furthermore, they are an ecofriendly and sustainable additive because they possess natural biodegradable characteristics and can decrease the application of chemicals. Plant essential oils can replace the antibiotic as feed additives due to their inhibition on bacteria and fungi. With the widely application of plant essential oils, their effects on improving the silage quality have been studied. Chen et al. (2023) reviewed the effects of essential oils and their activity on inhibiting bacteria and fungi, and explored the contribution of plant essential oils to silage quality, and provided some knowledge to the application and development of plant essential oils as feed additives in silage production. They found that plant essential oils can inhibit the growth and activity of various harmful microorganisms in silages whereby their ability to affect the permeability of cell membrane, ATPase activity and cell division, and limit biofilm formation. The application of suitable doses of plant essential oils in producing silage could affect microbial compositions via limiting the growth of some undesirable microbes such as yeast, Fusarium and Clostridium in silages, and indirectly accelerate LAB strains to become predominant microbes, decrease nutritional loss in silages, enhance aerobic stability and fermentation quality of silages.

With the rapid enhancement of global population and economy, the demand for animal products like milk, egg and meat are increasing. The shortage of feeds in animal industry is a global issue limiting the development of livestock. Natural woody plants can be found in many districts with a big biomass yield. The fresh branches and leaves of many woody plants have high levels of nutritional compositions like amino acids, crude protein, minerals and vitamins, and could be utilized to produce silages for animals. Hence, it is important to develop and use the woody plants for producing the clean and sustainable feeds in livestock. Du et al. (2023) reviewed the research progress, current status and development prospects of natural woody plant feed resources. The nutritive value and application of natural woody plants, the main factors influencing the silage quality of woody plant and the interaction mechanisms between secondary metabolites and microbial co-occurrence network were studied. Different preparing technologies for clean fermentation of woody plant silages were summarized, which presented a sustainable way for enhancing the production efficiency of livestock. Hence, woody plants are very important as a potential source of natural feeds in reducing feed shortage and accelerating sustainable development of livestock products.

In summary, this Research Topic explored the preharvest and postharvest factors influencing forage quality, physiology, and fermentation characteristics using various approaches. This Research Topic provided interesting results, and provided solution to some unresolved issues that need more exploration in producing silage. However, there is still a deep study gap identifying the impact of preharvest and postharvest factors on animal response. Therefore, further studies should focus on improving the field management and harvesting methods in silage production and in relation to animal performance.

## Author contributions

SW: Conceptualization, Data curation, Formal analysis, Funding acquisition, Investigation, Methodology, Project administration, Resources, Software, Supervision, Validation, Visualization, Writing – original draft, Writing – review & editing. QZ: Data curation, Methodology, Supervision, Writing – review & editing. LS: Formal analysis, Project administration, Validation, Writing – review & editing. HP: Data curation, Formal analysis, Methodology, Supervision, Writing – review & editing. PL: Formal analysis, Project administration, Validation, Writing – review & editing. NK: Data curation, Formal analysis, Methodology, Project administration, Resources, Supervision, Validation, Visualization, Writing – review & editing.

